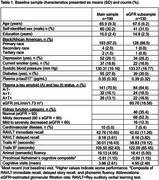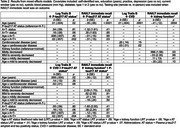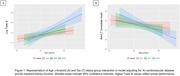# Plasma *p*‐tau217 A+T+ status is associated with accelerated cognitive decline in the African Americans Fighting Alzheimer's in Midlife study

**DOI:** 10.1002/alz70856_106979

**Published:** 2026-01-08

**Authors:** Gilda E. Ennis, Derek L. Norton, Rebecca E. Langhough, Diane Carol Gooding, Megan L. Zuelsdorff, Shenikqua Bouges, Sanjay Asthana, Sterling C Johnson, Henrik Zetterberg, Carey E. Gleason

**Affiliations:** ^1^ Wisconsin Alzheimer's Disease Research Center, University of Wisconsin School of Medicine and Public Health, Madison, WI, USA; ^2^ Department of Psychiatry, University of Wisconsin‐Madison, School of Medicine and Public Health, Madison, WI, USA; ^3^ School of Nursing, University of Wisconsin‐ Madison, Madison, WI, USA; ^4^ Wisconsin Alzheimer's Disease Research Center, School of Medicine and Public Health, University of Wisconsin‐Madison, Madison, WI, USA; ^5^ Institute of Neuroscience and Physiology, the Sahlgrenska Academy at the University of Gothenburg, Gothenburg, Sweden; ^6^ Wisconsin Alzheimer's Disease Research Center, School of Medicine and Public Health, University of Wisconsin‐Madison, Madison, WI, USA; ^7^ University of Wisconsin School of Medicine and Public Health, Madison, WI, USA

## Abstract

**Background:**

Higher plasma *p*‐tau217 levels have been related to cognitive decline in predominantly European American and European adult samples. Some studies suggest cardiovascular disease (CVD) and kidney disease, comorbidities more common in African American (AA) adults, are associated with cognitive dysfunction and elevated *p*‐tau217; consequently, these comorbidities may confound associations between *p*‐tau217 and cognition. Using *p*‐tau217 thresholds for amyloid (A) and tau (T) positive status, we examined if AT status associated with cognitive decline in an AA sample and if significant associations persisted when adjusting for comorbidities also identified as significant predictors of worsening cognition.

**Method:**

*N* = 199 African Americans Fighting Alzheimer's in Midlife (AA‐FAIM) study participants, without baseline dementia, had cognitive data and plasma (EDTA) *p*‐tau217 (AlzPath, Inc.) for analysis (Table 1). *N* = 130 had baseline estimated‐glomerular‐filtration‐rate (eGFR) to assess kidney function. CVD was defined as baseline history of myocardial‐infarction, congestive‐heart‐failure, or stroke. *p*‐tau217 thresholds for amyloid‐ and tau‐positive status (A+>.35pg/mL; T+>.556pg/mL) were derived from previous receiver‐operating‐characteristic‐curve analyses of AA‐FAIM amyloid‐ and tau‐PET data. Separate mixed effects models tested whether AT status (A+T+, A+T‐, A‐T‐[reference]), eGFR, 3‐level kidney function variable (mild‐to‐severe, mild, normal[reference]), and CVD moderated relationships between age and decline in: Rey‐auditory‐verbal‐learning‐test (RAVLT: immediate‐ and delayed‐recall), log‐transformed Trails A and B, semantic fluency, and preclinical‐Alzheimer's‐cognitive‐composite. If AT status, CVD and/or kidney‐function were significantly related to same cognitive outcome, the association between AT status and cognition was re‐tested adjusting for the comorbidity and age*comorbidity interaction. See Table 2 for covariates.

**Result:**

A+T+ status was related to greater decline in log‐transformed Trails B and RAVLT immediate‐recall; CVD predicted greater decline in log‐transformed Trails B; association between mild‐to‐severely decreased kidney function and decline in RAVLT immediate‐recall was not significant (*p* = .10) (Table 2). AT status and comorbidities were not associated with other cognitive outcomes. A+T+ status was associated with greater declines in log‐transformed Trails B and RAVLT immediate‐recall when adjusting, respectively, CVD and impaired kidney function and their interactions with age (Table 2; Figure 1).

**Conclusion:**

In AA‐FAIM, plasma *p*‐tau217 A+T+ status was related to greater decline in executive function and immediate‐recall, even following comorbidity adjustment. Replication is needed in samples having larger proportions with medical comorbidities.